# Factors associated with false negative and false positive results of prostate-specific antigen (PSA) and the impact on patient health: Cohort study protocol

**DOI:** 10.1097/MD.0000000000017451

**Published:** 2019-10-04

**Authors:** Mari Carmen Bernal-Soriano, Lucy A. Parker, Maite López-Garrigos, Ildefonso Hernández-Aguado, Juan P. Caballero-Romeu, Luis Gómez-Pérez, Rocío Alfayate-Guerra, María Pastor-Valero, Nuria García, Blanca Lumbreras

**Affiliations:** a CIBER de Epidemiología y Salud Pública (CIBERESP), Public Health Department, Miguel Hernández University of Elche; b Clinical Laboratory Department, University Hospital of San Juan de Alicante; c Urology Department, University Hospital of Vinalopó, Alicante, Spain and Alicante Institute for Health and Biomedical Research (ISABIAL); d Urology Department, University Hospital of San Juan de Alicante, Alicante, Spain and Pathology and Surgery Department, Miguel Hernández University of Elche; e Clinical Laboratory Department; f Urology Department, University General Hospital of Alicante, Alicante, Spain.

**Keywords:** false negative reactions, false positive reactions, prostate disease, prostate-specific antigen, quality in health care

## Abstract

**Introduction:**

Prostate-specific antigen (PSA) is the main tool for early detection, risk stratification and monitoring of prostate cancer (PCa). However, there are controversies about the use of PSA as a population screening test because of the high potential for overdiagnosis and overtreatment associated. The net benefit of screening is unclear and according to the available recommendations, it should be offered to well-informed men with an adequate health status and a life-expectancy of at least 10 years or to men at elevated risk of having PCa. In addition, the factors that influence test results are unclear, as is impact of false positive or negative results on patient health.

Our objective is to assess the clinical and analytical factors associated with the presence of false positive and false negative results and the diagnostic/therapeutic process followed by these patients.

**Methods and analysis:**

A prospective observational cohort study will be carried out. We will include a cohort of patients with a positive PSA result (1.081 patients) and a sample of patients with negative results (572 patients); both will be followed for 2 years by reviewing medical records to assess the variables associated with these results, as well as characteristics of patient management after a positive PSA value. We will include those patients with a PSA determination from 2 hospitals in the Valencian Community. Patients who have been previously diagnosed with prostate cancer or who are being followed for previous high PSA values will be excluded.

**Discussion:**

The study will estimate the frequency of false positive and false negative PSA results in routine clinical practice, and allow us to quantify the potential harm caused.

**Study registration:**

Clinicaltrials.gov (https://clinicaltrials.gov/): NCT03978299, June 7, 2019.

## 1 Introduction

The number of cases of prostate cancer (PCa) has increased globally by 40% in 10 years, from 1.4 million in 2016 to 1.0 million in 2006.^[1]^ This increase has been associated with the introduction of Prostate Specific Antigen (PSA) testing in the 1990s, with the variation observed between countries explained by differences in use of PSA tests.^[2–4]^

The usefulness of PCa screening with PSA test in reducing mortality has been questioned for years due to contradictory results of two prospective randomized trials.^[5]^ Methodological limitations were observed in one of the trials, where more than 90% of men in the control group were screened anyway.^[6]^ A posterior analysis of both trials concluded that the benefit of screening in terms of PCa mortality reduction was similar in both (around 30%).^[7]^ In addition, an increase in the absolute effect of screening on PCa mortality has been described with longer follow-up.^[8]^ A retrospective cohort study has suggested that PSA screening has benefit in both PCa specific and overall mortality.^[9]^ While, a systematic review published in 2018 concludes that PCa screening leads to a small reduction in disease-specific mortality over 10 years but has no effect on overall mortality.^[10]^

Although PSA determination may help in the early detection of PCa, it has some limitations. First, it is not a specific PCa biomarker, as it can be high in other circumstances (such as acute prostatitis, benign prostatic hyperplasia, after catheter manipulations, etc). Second, PSA concentration is a continuous parameter, meaning that there is no universally accepted threshold value for PCa diagnosis, although normal values <4 μg/L are often used. Finally, it cannot detect the aggressiveness of tumors and can lead to overdiagnosis,^[11]^ meaning some cancers diagnosed by early detection develop so slowly that they may never cause the patient problems. It has been estimated that between 20 and 50% of PCa are overdiagnosed.^[12–14]^

There is no controversy about the harm resulting from PSA screening, such as consequences of overdiagnosis.^[14,15]^ Diagnoses associated not only with wasted resources and side effects of treatment, but also with severe, stress-induced psychiatric and somatic consequences.^[15]^ Therefore, various strategies have been studied to reduce these harms. A study that has evaluated low-intensity screening to reduce overdetection continued to deliver greater detection of low-risk PCa, without reducing mortality linked to PCa.^[16]^ While, active surveillance (AS) is a proposed serial monitoring program to reduce PCa overtreatment due to screening and avoid immediate therapy for patients with low-risk PCa.^[17]^ PCa mortality results described in patients with AS have been similar to low-risk patients with initial definitive intervention.^[18]^

Finally, the main North American and European professional associations provide a series of recommendations regarding opportunistic screening to reduce overdiagnosis. The U.S. Preventive Services Task Force (USPSTF) updated its recommendations in 2017, in which it indicated that opportunistic screening may be useful among men aged between 55 and 69 years of age but the decision to perform this test should be made by each patient individually and jointly with the clinician, after the patient understands the benefits and risks of screening.^[19]^ The European Association of Urology (EAU)^[20]^ recommends the doctors offer an individualized early detection strategy to informed patients with good performance status and life expectancy of at least 10 to 15 years with high risk of PCa (men older than 50 years or 45 years if they are African American or have a family history of PCa).

Furthermore, several clinical or sociodemographic factors may influence PSA levels, how results interpreted, and hence false positives or negatives in a PSA test. Previous studies have evaluated variations of PSA levels according to demographic parameters such as age,^[21]^ although the clinical significance of these variations has not been evaluated in clinical practice. Regarding race, there are studies that showed variations in PSA level^[22]^ while others found no differences.^[23]^ Some studies suggest that clinical factors such as diabetes^[24]^ and obesity^[25]^ are associated with a lower likelihood of having a positive PSA test. Similarly, treatments like statins,^[26]^ metformin^[27]^ or treatment for benign prostatic hypertrophy^[28]^ can also affect PSA results. Many of these factors are highly prevalent in the target population for PCa screening.

To date, there are no studies evaluating the impact of these factors on a false positive or negative PSA result performed in general practice. The majority of studies have been carried out as part of clinical trials that evaluate the usefulness of PSA as a screening test, where the population is different from that with a PSA determination in routine clinical practice. Clinical trials tend to include healthier and younger patients and often represent a highly selected patient population.^[29]^ The impact of PSA determination on the patient's clinical management and evolution has not been evaluated either. This includes possible adverse effects related to the diagnostic process (biopsy, surgery and treatment), and the relationship between PSA determination and other factors that are considered when making the decision to request a biopsy such as patient comorbidity, Charlson index and PSA density, index or velocity.

## 2 Objectives

### 2.1 Primary objective

The primary aim of the current research project is to evaluate the outcomes of PSA determinations, in general practice in two health departments of the Valencian Community (Spain).

### 2.2 Specific objectives

1. To analyze the clinical and analytical factors associated with the presence of false positive and false negative results in PSA determinations in patients undergoing opportunistic screening or with symptoms suggestive of disease.
2. To evaluate the patient's clinical outcome, and any diagnostic and/or clinical and/or therapeutic interventions over a 2-year period after PSA testing. Furthermore, we will study whether this management is appropriate to the recommendations of the European Association of Urology.

## 3 Methods and analysis

This study will be carried out and reported according to with the Strengthening the Reporting of Observational Studies in Epidemiology (STROBE) Statement.^[30]^

### 3.1 Study design

This is a prospective observational cohort study of patients with a PSA determination for the early detection of PCa or in the presence of prostatic symptoms, in the general practice. The study protocol is registered at https://clinicaltrials.gov (ClinicalTrials.gov Identifier: NCT03978299).

### 3.2 Study population

#### 3.2.1 Eligibility criteria

Participants will be a random sample of men from the Health Departments 17 and 19, in the Valencian Community (these include 20 primary health centers and 2 hospitals: General University Hospital of Sant Joan d’Alacant and General University Hospital of Alicante, respectively). These are referral hospitals for all individuals living in their catchment areas and belong to the National Health Care System (the majority of the population in Spain uses the National Health System (NHS) as the main medical service (the publicly funded insurance scheme covers 98.5% of the Spanish population). We will include men over 18 with a PSA determination requested in a routine health examination from January to April 2018. Patients who do not belong to the NHS or have been previously diagnosed with PCa or who are being followed for previous high PSA values will be excluded.

We will select a cohort of patients with positive PSA results (defined as total PSA value is >10 μg/L or a total PSA between 4 and 10 μg/L if the value of the free PSA/total PSA fraction is <25% in at least in two determinations) and a cohort of patients with negative results (defined as total PSA value is < 4 μg/L or a total PSA between 4 and 10 μg/L if the value of the free PSA/total PSA fraction is >25%) among subject included in a previous cross-sectional study (NCT03968692) that we are carrying out (registered in ClinicalTrials.gov (NCT03968692)). This cross-sectional study aims to describe the PSA determinations that are performed in clinical practice and their appropriateness according to the available recommendations, considering sociodemographic and clinical characteristics of the patients.

#### 3.2.2 Sample size and recruitment procedure

**Figure 1:**
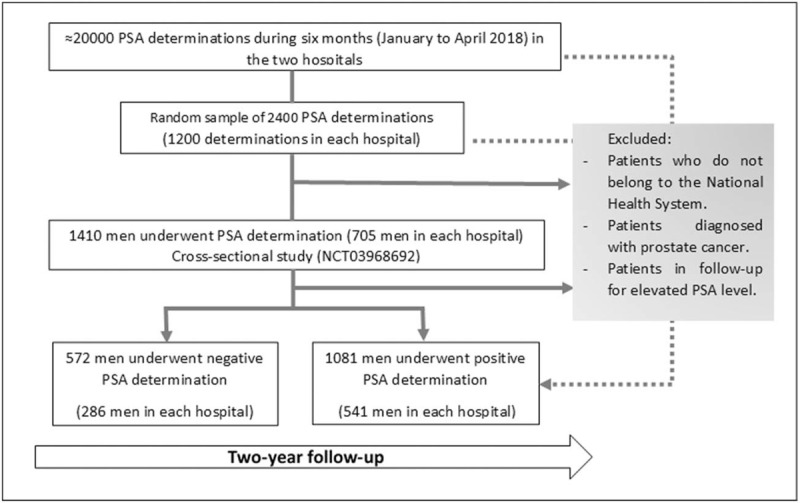
Patient selection and recruitment.

According to a review by the American Cancer Society,^[31]^ a value of PSA of 4 μg/L had an estimated sensitivity of 21% to detect any type of prostate cancer and a specificity of 91%. We estimate a prevalence of PCa in this population not lower than 5% (given that we include asymptomatic and symptomatic patients), with a 95% margin of error and 2% precision, we will need to include 457 patients with a negative PSA result and 865 PSA-positive patients. Taking into account a 20% possible loss during follow-up, we will increase to 572 patients with a negative PSA result (286 per center) and 1081 patients with a positive PSA result (541 per center) who will be selected consecutively from among those included in the previous cross-sectional study (NCT03968692) until we reach the proposed sample size. We will use the initial randomized list (which included determinations during the first 4 months of 2018) to select patients who meet both negative and positive PSA criteria and if it is necessary, we will continue to review analyses until the sample size is achieved. (Fig. 1)

### 3.3 Data collection procedure

Both hospitals have digital medical records which will be used to collect individual patient data. Furthermore, we will use the variables collected for the cross-sectional study as baseline data. These variables include demographic and clinical characteristics (patient who has the PSA determination as part of opportunistic screening or due to the presence of symptoms suggestive of disease), setting (primary care or clinical service), toxic habits, previous history of cancer, family history, present pharmacological treatment, PSA tests carried out in the last 12 months and PSA value, anthropometric measures and other comorbidities (Table 1).

**Table 1:**
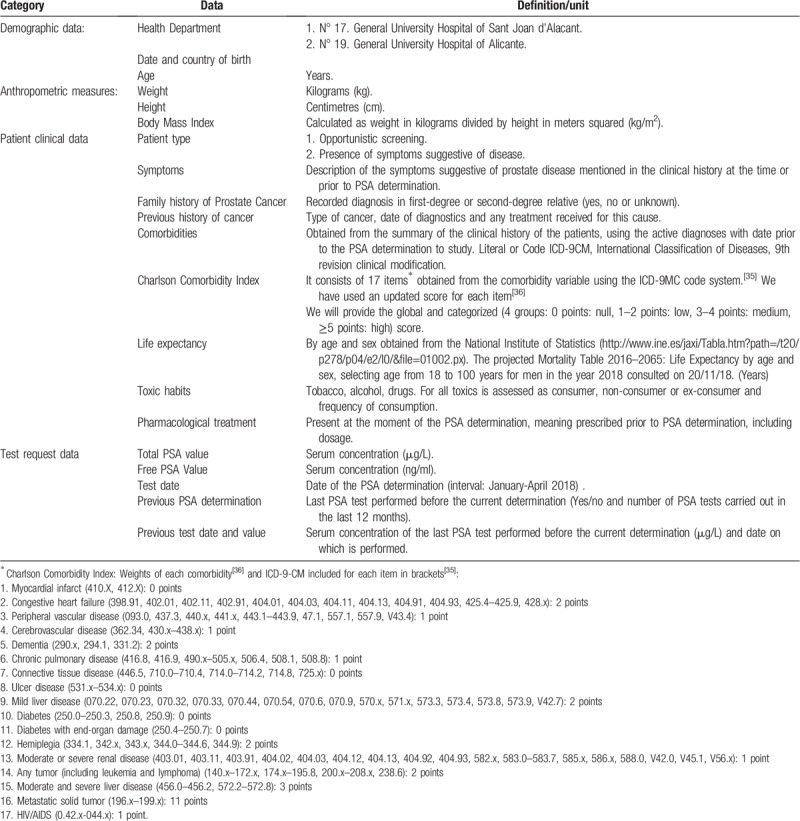
Information to be extracted from the medical history.

We will follow both cohorts (positive and negative PSA results) for 2 years by reviewing their medical records (every 3 months for patients with a positive PSA result and annually for patients with a negative result). The following variables will be recorded: diagnostic interventions (digital rectal exam, biopsy, PSA determination, imaging tests), clinical interventions (medical consultations) and therapeutic interventions (radiotherapy, surgery, chemotherapy, hormonal treatment) during follow-up, collecting for each intervention the date and result obtained; and cancer diagnosis (date and cancer type).

### 3.4 Outcomes

The study outcomes associated with specific objective 1 are:

1. The effect size of the associations between clinical and analytical factors and false positive result in the determination of serum PSA levels (defined as positive result: if the total PSA value is >10 μg/L or a total PSA between 4 and 10 μg/L if the value of the free PSA/total PSA fraction is <25% in at least in 2 determinations and the result of digital rectal examination(s) and/or subsequent biopsy or biopsies are negative, according to the latest recommendations of the European Association of Urology^[20]^).
2. The effect size of the associations between clinical and analytical factors and false negative result in the determination of serum PSA levels (defined as false negative result: if the PSA value is negative, and the patient is diagnosed with PCa in the subsequent follow-up, 2 years, according to the latest recommendations of the European Association of Urology^[20]^).

While for specific objective 2 the study outcomes are as follows:

1. Frequency of patients with appropriate diagnostic interventions performed in those with a positive serum PSA value according to “EAU - ESTRO - ESUR - SIOG Guidelines on Prostate Cancer”.^[32]^ Which is defined as strategies that satisfies or not satisfies EAU recommendations with regard to the following issues (Recommendations for clinical diagnosis of prostate cancer and Recommendations for repeat-biopsy imaging)
2. Frequency of prostate cancer patients with an appropriate therapeutic interventions according to “EAU - ESTRO - ESUR - SIOG Guidelines on Prostate Cancer”.^[32]^ Which is defined according to the EAU recommendations with regard to the number and type of therapeutic interventions carried out after prostate cancer diagnosis. The following information will be considered: surgical treatments, radiotherapeutic treatments, active therapeutic options outside surgery and radiotherapy.

Two researchers will independently analyze diagnostic, and therapeutic interventions performed after a positive PSA value the follow-up deadline, and their adaptation to the latest available recommendations.^[32]^ Each patient will be classified as “Appropriate according to guideline”, “more intensive than guideline” or “less intensive than guideline”.

### 3.5 Statistical analysis

The analysis will be performed using the Stata IC 15 program.

- Specific objective 1. We will calculate the proportion of false positive and false negative results for the diagnosis of PCa and the associated variables. In the case of false positive tests, we will also describe the time from a positive PSA result until PCa is ruled out using mean, standard deviation or median and interquartile ranges for the total population and relevant subgroups. In order to analyze clinical and analytical factors associated with the presence of false positive and false negative results in PSA determinations, we will calculate prevalence ratios and their 95% confidence interval with log-binomial regression.
- Specific objective 2. We will assess the probability of having a diagnostic, surgical or therapeutic intervention according to variables using risk ratios with their 95% confidence intervals. If necessary, we will adjust for potential confounders using log-binomial regression. We will also evaluate the agreement between the interventions performed and the available guidelines.^[20]^ We will evaluate the inter-observer agreement in the determination of appropriateness using the Kappa index.

Steps will be taken to prevent missing data (e.g., we will periodically review medical records and access data from different sources such as records of primary, specialized and hospital care), but some level is unavoidable and we will incorporate methods analyzing missing data or data from uncertain sources when necessary.^[33,34]^

### 3.6 Ethics approval and consent to participate

This study has been approved (17/324) by Clinical Research Ethics Committee (CEIC) of the Hospital Sant Joan d’Alacant. As data will only be collected from the clinical history of patients (with a sample size of approximately 3000 subjects), we considered it unfeasible to obtain the informed consent of patients, without large losses of cases and significant selection biases. For this reason, and due the absence of any significant risk to the patients from their records being accessed, the CEIC approved a waiver of the informed consent requirement. In the research database, patients will be anonymized using dissociated codes, unidentifiable, meaningless to any other information system and which will not allow the identification of individual patients or their crossing with other databases. Since the project database will not contain any data that would allow the identification of patients, no declaration to the Data Protection Agency is required.

### 3.7 Dissemination

The results of the study will be published in peer-reviewed journals and presented at congresses. In addition, we will distribute a report with detailed results and an assessment of their impact on current recommendations to technology assessment agencies, and will be presented at national meetings of urology and laboratory medicine societies.

Some of the research staff belongs to the Clinical Epidemiology research group within Centro de Investigación Biomédica en Red de Epidemiología y Salud Pública (CIBERESP), and one of the initiatives of this group is the MAPAC project (Improvement of the Adequacy of the Care and Clinical Process). This project has led to the organization of the MAPAC intra-hospital commissions, with the institutional collaboration of the Hospital Management Directorates of the groups attached to the program. It will facilitate the incorporation of the protocol of action resulting from this study in other health centers, and the establishment of synergies between different professionals. Regarding implementation of prostate cancer screening recommendation in clinical care, a member of the research team (IHA) is engaged with high level assessment to health authorities, both at regional and national level and will present the results to target decision makers’ audience.

## 4 Discussion

The present study will allow showing the proportion of false positives and negatives obtained in clinical practice. We will provide knowledge on the modification of PSA levels according to relevant clinical variables and the association of the value of PSA with diagnostic and clinical interventions. This may support the basis of new recommendations about relevant aspects of urological clinical such as: the predictive value of clinical/diagnostic interventions associated with PSA value and patient management, depending on patient characteristics and the value of PSA and other clinical/diagnostic interventions obtained.

This study is not without limitations. First, we will retrieve the data from medical files, thus the quality of the data collected is highly dependent on the quality of the information recorded in the files. Thankfully, a recent update to an electronic system in the participating hospitals makes is possible for us to access data from different sources (primary, specialized and hospital care) and this is likely to improve data completeness and quality for the study. In addition, the UAE recommends using the Geriatric-8 and mini-COG tools for health status screening.^[32]^ We will not have all the information to apply them and instead we use life expectancy together with the Charlson Comorbidity Index.

Second, some degree of loss to follow-up is unavoidable and this may impact study results. Periodic review of the medical records should minimize this limitation. Finally, due to the nature of the study, we will not be able to evaluate the presence of overdiagnosis and overtreatment. We plan to evaluate the stage and characteristics of the PSA-detected prostate tumors, which may give us an indication of the potential for overdiagnosis, but given that the study is carried out in a routine clinical population, it is not possible to include a control population.

## Author contributions

**Conceptualization:** Mari Carmen Bernal-Soriano, Lucy A. Parker, Maite López Garrigos, Ildefonso Hernández-Aguado, Juan Pablo Caballero Romeu, Luis Gómez Pérez, Rocío Alfayate Guerra, María Pastor Valero, Nuria García, Blanca Lumbreras.

**Funding acquisition:** Blanca Lumbreras.

**Investigation:** Mari Carmen Bernal-Soriano, Lucy A. Parker, Maite López Garrigos, Ildefonso Hernández-Aguado, Juan Pablo Caballero Romeu, Luis Gómez Pérez, María Pastor Valero, Nuria García, Blanca Lumbreras.

**Methodology:** Mari Carmen Bernal-Soriano, Lucy A. Parker, Maite López Garrigos, Ildefonso Hernández-Aguado, Juan Pablo Caballero Romeu, Luis Gómez Pérez, Rocío Alfayate Guerra, María Pastor Valero, Nuria García, Blanca Lumbreras.

**Supervision:** Lucy A. Parker.

**Writing – original draft:** Mari Carmen Bernal-Soriano, Lucy A. Parker, Maite López Garrigos, Ildefonso Hernández-Aguado, Juan Pablo Caballero Romeu, Luis Gómez Pérez, Rocío Alfayate Guerra, María Pastor Valero, Nuria García, Blanca Lumbreras.

**Writing – review & editing:** Mari Carmen Bernal-Soriano, Lucy A. Parker, Maite López Garrigos, Ildefonso Hernández-Aguado, Juan Pablo Caballero Romeu, Luis Gómez Pérez, Rocío Alfayate Guerra, María Pastor Valero, Nuria García, Blanca Lumbreras.

Mari Carmen Bernal-Soriano orcid: 0000-0002-9174-0068.
